# A Double-Primary Dead-Weight Tester for Pressures (35–175) kPa in Gage Mode

**DOI:** 10.6028/jres.108.003

**Published:** 2003-02-01

**Authors:** Kamlesh Jain, Yueqin Cen, Walter J. Bowers, James W. Schmidt

**Affiliations:** National Institute of Standards and Technology, Gaithersburg, MD 20899-0001

**Keywords:** dead-weight tester, piston/cylinder assembly, pistongage, pressure measurement, primary pressure standards

## Abstract

Primary pressure standards in the atmospheric pressure range are often established using mercury manometers. Less frequently, controlled-clearance dead-weight testers in which one component (normally the piston) has been dimensionally measured have also been used. Recent advances in technology on two fronts i) the fabrication of large-diameter pistons and cylinders with good geometry; and ii) the ability to measure the dimensions of these components, have allowed some dead-weight testers at NIST to approach total relative uncertainties (*k* = 2) in dimensionally-derived effective areas near 5 × 10^−6^. This paper describes a single piston/cylinder assembly (NIST-PG201WC/WC) that serves as both a primary gage in which both piston and cylinder are measured dimensionally and a controlled-clearance primary gage (employing the Heydemann-Welch method). Thus it allows some previous assumptions about the modeling of dead-weight testers to be checked. For the gage described in this paper the piston/cylinder clearance obtained from the two analyses have relative differences of 4 × 10^−6^ to 7 × 10^−6^ over the pressure range 35 kPa to 175 kPa. Some implications of these results will be discussed. From the dimensional characterizations and auxiliary measurements we have determined that the effective area for this gauge at 20 °C is:

$$A_{\text{eff},20}=1961.0659{\text{mm}}^{2}(1+3.75\times 10^{-12}P/\text{Pa}+3.05\times 10^{-12}P_{J}/\text{Pa}),$$

where *P* is the system pressure and *P*_J_ is a control pressure. The estimated relative uncertainty in effective area is 8.2 × 10^−6^ +1.4 × 10^−11^ *P*/Pa (*k* = 2). The temperature coefficient for the area was measured and found to be (9.06 ± 0.04) × 10^−6^/K. Thus using the gage at a reference temperature of 23 °C yields an effective area:

$$A_{\text{eff},23}=1961.1192{\text{mm}}^{2}(1+3.75\times 10^{-12}P/\text{Pa}+3.05\times 10^{-12}P_{J}/\text{Pa}),$$

with almost no increase in the uncertainty over that at 20 °C.

## 1. Introduction

The primary pressure standards in the atmospheric pressure range at the National Institute of Standards and Technology (NIST) and at the National Physical Laboratory-India (NPL-I) are presently established using mercury manometers [1–4]. However, recent developments in the fabrication of large-diameter high-quality pistons/cylinder assemblies and in dimensional metrology has allowed the pressure measurement community to contemplate primary pressure standards that are based on pistons and cylinders whose uncertainties could approach the best manometers.

The Pressure and Vacuum Group at NIST has recently acquired a new generation of piston/cylinder assemblies that operate in the 30 kPa to 175 kPa pressure range. The new gages have large diameters (~50 mm), which allow the diameter of each piece to be determined with a total uncertainty less than 50 nm (*k* = 1). This measurement uncertainty, in particular of the cylinder (typically more difficult), allows a more direct determination of the effective areas of these gages. In favorable cases this uncertainty could lead to pressures with relative standard uncertainty u(*P*)/*P* as low as (2 to 2½) × 10^−6^.

These same artifacts are also designed so that the controlled-clearance method, invented by Johnson and Newhall[5] and described by Heydemann and Welch[6] can be used to obtain an independent determination of the clearance between the piston and cylinder, *h/R*. Here *h* is the clearance width and *R* is the radius of the gage.

We find that the two determinations (direct dimensional vs controlled clearance) of *h/R*, differ from each other in relative effective area by about 4.6 × 10^−6^ to 8 × 10^−6^ over the range 35 kPa to 175 kPa. The dimensional characterization has an estimated relative standard uncertainty of 2.3 × 10^−6^ while the Heydemann Welch (H-W) characterization has an estimated relative standard uncertainty of 3.9 × 10^−6^.

One possibility for the difference between the two characterizations at high pressure is that the H-W characterization may not give the best results in cases in which one of the components is not perfectly round.

In the next sections we give a brief description of the apparatus and follow that with details of both characterizations. One characterization uses a dimensional measurement with an estimate of the pressure coefficient based on elasticity theory to obtain the effective area. The other uses a dimensional measurement of either the piston or the cylinder together with the H-W method to obtain the effective area.

## 2. Apparatus

For the present measurements we used a dead-weight tester with a large (50 mm diameter) piston/cylinder assembly made by DHI^1^. Both piston and cylinder were made of tungsten–carbide. Nominal values for Young’s modulus, *E*, and Poisson’s ratio, *ν*, were used to calculate the pressure coefficient [7]. Speed-of-sound measurements are planned in order to obtain lower uncertainties for *E* and *ν*, but because the intended pressure range is not large for the present apparatus, nominal values are adequate for estimating the pressure coefficient of the gage.

**Fig. 1:**
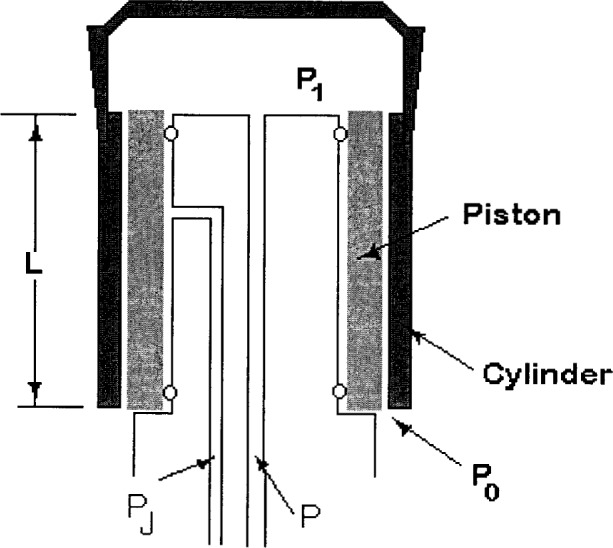
Schematic representation of the 50 mm piston/cylinder assembly. The lighter region represents the stationary piston while the darker region represents the floating cylinder. The size of the piston can be expanded with the application of a control pressure *P*_J_.

The assembly uses a floating cylinder design rather than the more usual floating piston design. (See Fig. 1.) An important feature of this design for the present measurements is that the diameter of the piston, or stationary element, can be controlled with the application of an independent control pressure. Thus the controlled-clearance method can be employed to determine the clearance between piston and cylinder [5–6] and independently to compare the clearance with direct dimensional measurements. At present a partial set of dimensional measurements on the piston/cylinder assembly is available from NIST. Other complementary measurements were made by NPL-I.

Other apparatus was used for two auxiliary measurements; a) the measurement of the thermal expansion coefficient, and b) a capacitance based measurement of the piston/cylinder. A temperature controlled environmental chamber (oven/cooler) was constructed for the 50 mm piston/cylinder (P/C) assembly and base, and was used in the measurement of the thermal expansion coefficient of the P/C assembly. A reference P/C assembly was used to monitor the pressure generated by the 50 mm P/C assembly in the chamber. The chamber was capable of ± 0.005 K stability (*k* = 1). The temperature of the chamber could be controlled between 0 °C and 50 °C and could be measured with a calibrated thermometer to better than ± 0.01 K (*k* = 1). With the P/C assembly inside, however, the chamber was operated only between 15 °C and 35 °C in order to avoid possible damage to the P/C assembly. In general, a wider temperature span yields a more accurate expansion coefficient. The thermal expansion coefficient for the piston/cylinder assembly’s area was found to be α = (9.06 ± 0.04) × 10^−6^/K (*k* = 1).

A capacitance gauge with ± 0.1 nF resolution was used to measure the capacitance between the piston and cylinder. One electrode was attached to the base of the assembly at ground potential. The other electrode was connected to the cylinder through a small cup that contained a tiny amount of mercury in order to minimize extraneous non-axial forces on the cylinder assembly. Minimal efforts were made to shield extraneous signals from the capacitance gauge. Values for the capacitance ranged between 100 nFd and 160 nFd.

## 3. Characterization from Dimensional Measurements

The Precision Engineering Division at NIST measured the dimensions of the piston and cylinder. Diameters were measured along two directrices (two longitudes, 0° to 180° and 90° to 270°) for both pieces. For the piston, a full set of roundness and straightness data was obtained [9a], and diameters were obtained at three places in both vertical planes. For the cylinder, diameters were obtained at 10 places along the two vertical planes [9b]. Diameters were measured at 20 °C.

The diameters were averaged for both piston and cylinder, and this yielded values for the areas of each component at 20 °C.

$$A_{0p,20}=\pi {D_{p}}^{2}/4\sim \pi {\left(49.96870\text{mm}\right)}^{2}/4,$$ (1a)

and

$$A_{0c,20}=\pi {D_{c}}^{2}/4\sim \pi {\left(49.96941\text{mm}\right)}^{2}/4.$$ (1b)

Here *D*_p_ and *D*_c_ are the average diameters of the piston and cylinder, respectively. The zero pressure effective area of the assembly derived from these measurements and adjusted to a reference temperature of 23 °C is:

$$\begin{array}{l} A_{0,23}=[(A_{0p,20}+A_{0c,20})/2][1+\alpha (T_{23}-T_{20})]\\ \;=(1961.1192\pm 0.0041){\text{mm}}^{2}. \end{array}$$ (2)

The uncertainty listed represents a combined relative uncertainty of 2.3 × 10^−6^ (*k* = 1). The measurements from NPL-I produced an effective area of (1961.155 ± 0.039) mm^2^ (*k* = 1) [8]; or a difference of about 0.035 mm^2^, which is within the combined uncertainty of the two measurements.

To obtain the effective area for the gage at higher pressures it is sufficient to obtain the pressure coefficients from nominal values of Young’s modulus and Poisson’s ratio. In this case the pressure coefficients for both piston and cylinder were derived from elasticity theory using the thick-wall formula[7].

The area of the piston given as a function of the two variables, generated pressure, *P*, and jacket pressure, *P*_J_, is:

$$A_{p}(P,P_{J})=A_{0p}(1+b_{p}P+b_{J}P_{J}),$$ (3a)

where

$$b_{p}=(-3.62\pm 0.18)\times 10^{-12}{\text{Pa}}^{-1}(k=1),$$ (3b)

and

$$b_{J}=(6.10\pm 0.3)\times 10^{-12}{\text{Pa}}^{-1}(k=1).$$ (3c)

Here, *b*_p_ is the pressure coefficient for the piston and it indicates the influence on the piston’s area by the generated pressure, *P*. Notice, that the formula for the area of the piston contains an additional pressure coefficient, *b*_J_, because of the unique design in which the piston’s area can be controlled with an auxiliary control pressure *P*_J_. [Normally the subscript “J” would refer to a “jacket” or “external” pressure. With the present design the control pressure is placed on the inside of the piston rather than the outside of the cylinder. We prefer to continue to use the subscript “J” instead of “cc” to avoid confusion with the cylinder‘s pressure coefficient, which we will denote as *b*_c_. (See below.)]

The area of the cylinder as a function of generated pressure is:

$$A_{c}(P)=A_{0c}(1+b_{c}P),$$ (4a)

where

$$b_{c}=(11.12\pm 0.55)\times 10^{-12}{\text{Pa}}^{-1}.$$ (4b)

The effective area for the gauge at *T* = 23 °C is given by:

$$A(P,P_{J})=A_{0,23}\{1+[(b_{p}+b_{c})/2]P+(b_{J}/2)P_{J}\},$$ (5a)

$$A(P,P_{J})=1961.1192{\text{mm}}^{2}\times (1+3.75\times 10^{-12}P/\text{Pa}+3.05\times 10^{-12}P_{J}/\text{Pa}).$$ (5b)

**Fig. 2:**
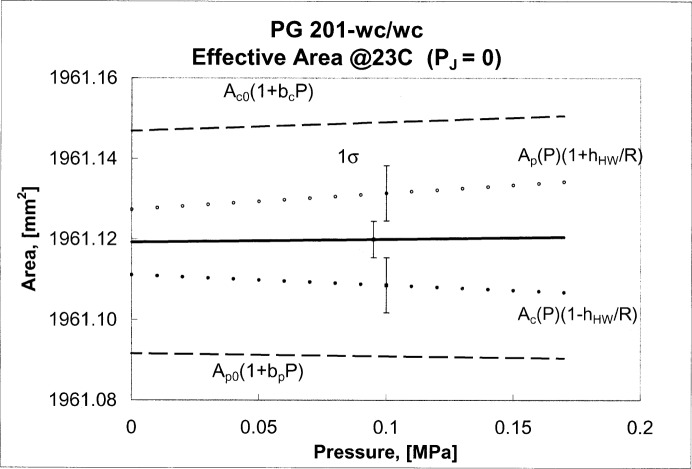
Effective area of the 50 mm piston/cylinder assembly (PG 201-WC/WC) obtained using two methods. The heavy solid line represents the area obtained from dimensional measurements together with a calculated pressure coefficient. The dotted lines represent the areas obtained from the Heydemann-Welch method. The two dashed lines represent the dimensionally obtained areas of the piston and cylinder taken individually.

The effective area from Eq. (5) is plotted in Fig. 2 as the solid line. The separate dimensionally measured areas of the piston and cylinder are shown as the dashed lines (Eqs. (3) and (4), respectively).

For the cylinder a complete-set of roundness/straightness data were not available from NIST. But the diameters were measured at enough places to suggest an out of roundness condition for the cylinder of about 900 nm. These diameters provided motivation to obtain roundness and straightness data elsewhere and a set was subsequently obtained from NPL-I. The NPL-I data suggested that both piston and cylinder had peak to valley roundness deviations of at most + 100 nm with a combined standard uncertainty of ± 100 nm [8]. However, subsequent dimensional measurements (a repetition of diameters) at NIST seemed to confirm again the presence of the out-of-roundness condition.

## 4. Clearance Between Piston and Cylinder

### 4.1 Via Heydemann-Welch Method

The Heydemann-Welch method [5,6] can be used to estimate the clearance betwee the piston and cylinder, *h*:

$$h/R=-d(P_{z}-P_{J}),$$ (6)

where *h* is the estimated clearance, *R* is the radius of the gage, *d* and *P*_Z_ are parameters obtained in accordance with Ref. [6] and summarized below and *P*_J_ is the control pressure as indicated before. The value for *h/R* or “−d(*P*_Z_ − *P*_J_)” is then used in the formulas either to add the clearance to the piston’s area or subtract the clearance from the cylinder’s area to obtain the effective area of the gauge.

$$A_{+}(P,P_{J})=A_{0p}(1+b_{p}P+b_{J}P_{J})(1+|d(P_{Z}-P_{J})|),$$ (7)

$$A_{-}(P,P_{J})=A_{0c}(1+b_{c}P)(1-|d(P_{Z}-P_{J})|).$$ (8)

**Fig. 3:**
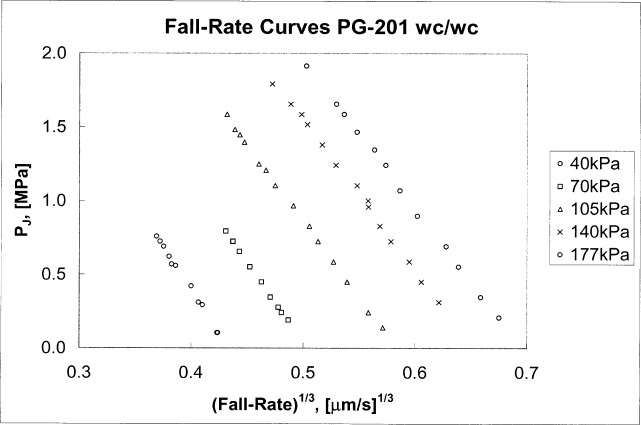
Control pressures *P*_J_ plotted against fall rates for several nominal loads, (*P*_1_ - *P*_0_), listed in the legend.

**Fig. 4:**
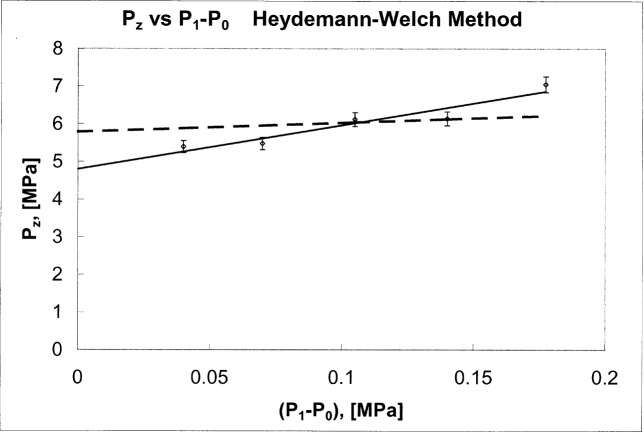
Control pressure intercepts at zero fall rate. (*P*_1_-*P*_0_) is the pressure generated by the gage. In gage mode *P*_0_ is the ambient pressure and approximately equal to 10^5^ Pa. The solid line is a fit to the extrapolated intercepts. The dashed line is a fit in which the slope is constrained to the theoretical value from elasticity theory.

In brief, *P*_Z_ is obtained from fall-rate measurements and represents the control pressure at which the crevice between the piston and cylinder would close. The fall-rate measurements plotted in Fig. 3 are listed in Table 1. Straight lines were fitted to each of the five load lines and extrapolated to the expected zero fall-rate intercept. Those intercepts are plotted in Fig. 4. The average value of the intercepts, *P*_Z_, (plotted in Fig. 4) and their standard deviation were found to be:

$$<P_{Z}>=(6.03\pm 0.82)\times 10^{6}\text{Pa}.$$ (9)

The zero-fall rate intercepts indicate the presence of a possible pressure dependence for *P*_z_(*P*). Fitting the intercepts to a straight line gives:

$$P_{Z,\text{meas}}(P)=(4.8\pm 0.2)\times 10^{6}\text{Pa}+(11.6\pm 2.1)P,$$ (10)

and is represented by the solid line in Fig. 4. The slope can also be estimated from elasticity theory and is about 2.4 (± 10 %). The dashed line plotted in Fig. 4 represents the result based on elasticity theory,

$$P_{Z,\text{el}}(P)=(5.8\pm 0.2)\times 10^{6}\text{Pa}+(2.4\pm 0.12)P.$$ (11)

The uncertainties in slope were estimated from our knowledge of the material properties of the tungsten carbide and the geometry of the pieces involved using conventional formulae for stresses in cylinders. Since the two values for *P*_Z_ appear to differ, we choose to take a conservative approach and represent *P*_Z_ by the average of Eqs. (10) and (11) and to represent the uncertainty with a square distribution:

$$P_{Z}(P)=(5.3\pm 0.3)\times 10^{6}\text{Pa}+(7.0\pm 2.7)P.$$ (12)

The H-W parameter “*d*,”defined as *d* = (−1/*A*) d*A*/d*P*_J_, can be measured using a separate sufficiently sensitive pressure gauge. The data are listed in Table 2 and yield a measured average value:

$$d_{\text{meas}}=(-3.84\pm 0.03)\times 10^{-12}{\text{Pa}}^{-1}.$$ (13)

The value estimated from elasticity theory is:

$$d_{\text{el}}=(-3.03\pm 0.15)\times 10^{-12}{\text{Pa}}^{-1}.$$ (14)

The uncertainty in *d*_el_ was again estimated from our knowledge of the material properties of the tungsten carbide and the geometry of the pieces involved using conventional formulae for stresses in cylinders. We choose to represent “*d* ” by the average of Eqs. (13) and (14) and to represent the uncertainty with a square distribution:

$$d=(-3.44\pm 0.30)\times 10^{-12}{\text{Pa}}^{-1}.$$ (15)

The effective areas from Eqs. (7) and (8) are then:

$$A_{+}(P,P_{J})=A_{0p}(1-3.62\times 10^{-12}P/\text{Pa}+6.10\times 10^{-12}P_{J}/\text{Pa})\times [1+3.44\times 10^{-12}{\text{Pa}}^{-1}(5.3\times 10^{6}\text{Pa}+7.0P-P_{J})],$$ (16)

and

$$A\text{\_}(P,P_{J})=A_{0c}(1+11.12\times 10^{-12}P/\text{Pa})\times [1-3.44\times 10^{-12}{\text{Pa}}^{-1}(5.3\times 10^{6}\text{Pa}+7.0P-P_{J})].$$ (17)

The effective areas are plotted as the dotted lines in Fig. 2 for the case *P*_J_ = 0. The two areas *A*_−_ and *A*_+_ differ relatively by about 8 × 10^−6^ to 14 × 10^−6^. This difference could be a result of the inadequacy of the H-W model to accurately describe non-ideal (non-circular) geometries.

We reemphasize before proceeding to the next section that the H-W method for determining the area of a controlled-clearance gauge is, in effect, a method for estimating the clearance between the piston and cylinder. In the next section we will present results from two other methods for determining the clearance.

### 4.2 Auxiliary Clearance Measurements

The clearance, *h*, between the piston and cylinder can be determined using a variety of techniques and, although they do not provide direct help in reducing the uncertainty of the effective area, these measurements can provide consistency checks on the dimensional measurements and inferences based on these measurements. We have already mentioned two techniques: 1) Direct dimensional measurements and 2) fall-rate data coupled with “*d* ” measurements interpreted with the H-W formulation and dimensional measurements.

**Fig. 5:**
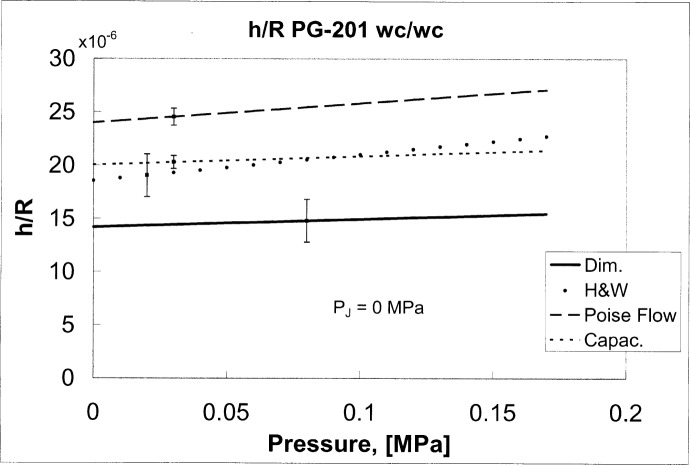
The clearance width can be estimated using a variety of independent methods, namely 1) direct dimensional measurements, 2) the H-W method, 3) the Poiseuille flow model and 4) capacitance measurements.

1. The dimensional measurements lead to:
  $$h_{\text{Dim}}(P,P_{J})=[(D_{c}-D_{p})/2][1+(b_{c}-b_{p})P-b_{J}P_{J}],$$ (18)
  where *h*_Dim_(*P,P*_J_) is the clearance as a function of *P* and *P*_J_. The average diameters *D*_c_ and *D*_p_ were determined from direct dimensional measurements at ambient pressure and *b*_c_, *b*_p_, *b*_J_ are pressure coefficients obtained from elasticity theory.
2. The fall-rate measurements interpreted with the aid of the H-W formulation give:
  $$h_{\text{HW}}(P,P_{J})=-R\times d[P_{Z}(P)-P_{J}),$$ (19)
  where *h*_HW_(*P,P*_J_) is the clearance based on the H-W model with parameters “*d* ” and *P*_Z_. *R* is the radius of the gauge. *h*_Dim_(*P,P*_J_)/*R* and *h*_HW_(*P,P*_J_)/*R* are plotted in Fig. 5 as the solid and dotted lines respectively for the case *P*_J_ = 0.
3. Fall-Rate measurements, interpreted with the Poiseuille flow equation for a uniform crevice [10,11], were also used to obtain the clearance:
  $$h_{\text{Poise}}={\left[12\frac{RP_{1}\eta L}{\left(P_{1}^{2}-P_{0}^{2}\right)}\times \frac{dz}{dt}\right]}^{1/3}.$$ (20)
  Here *η* (=1.786 × 10^−5^ Pa·s) is the viscosity of the pressure fluid (nitrogen), *L* (= 0.05 m) is the engagement length, *P*_0_ and *P*_1_ are the absolute pressures at the bottom and top of the crevice respectively and d*z*/d*t* is the fall rate which depends on P and *P*_J_. This method has been used by Molinar and Vatasso[12], by Dolinskii et al.[13] and by Meyers and Jessup [14]. The clearances obtained from Eq. (20) were fitted with an equation, linear in pressure, and this equation is plotted for the case *P*_J_ = 0 in Fig. 5 as the dashed line. This plot is based on the same fall-rate measurements used to determine *h*_HW_/*R*. Thus a comparison of *h*_Pois_/*R* with *h*_HW_/*R* is a comparison of the two models’ estimates for the same quantity, based in part on the same fall-rate data. The two models differ relatively by about 5 × 10^−6^ as shown in Fig. 5.
4. Lastly, clearances were determined using capacitance measurements[15]:
  $$h_{\text{cap}}=\varepsilon_{0}K\frac{2\pi RL}{C(P,P_{J})}.$$ (21)
  Here *ε*_0_ is the permittivity of the vacuum, *K* is the dielectric coefficient of the pressure fluid (nitrogen), and *C*(*P,P*_J_) is the measured capacitance, which changes depending on the pressure and jacket pressure. For the interpretation of the capacitance measurements an ideal geometry was assumed, as was the case for the interpretations of the fall-rate measurements using the Poiseuille flow model. The capacitance measurements appeared to be much more stable and linear when compared with the fall-rate measurements, which can vary significantly with ambient temperature changes. The clearances obtained from the capacitance measurements and Eq. (21) were also fitted by an equation linear in pressure. The residual variance from the fit was less than 2 nm as compared with the 10 nm residual variance for the fall-rate measurements. *h*_cap_/*R* is plotted in Fig. 5 as the dashed line for the case *P*_J_ = 0.

Note that the differences between the various measurements are considerable, in some cases more than twice the standard uncertainties. No single method gives the “true” measurement for the clearance needed to define the effective area. Each method measures its own average or “moment” of the distribution of clearances, *h* (*ϕ*, *z*). For an ideal case in which the clearance was absolutely uniform [*h*(**ϕ**,z) = H], then the different measurements would be expected to yield the same result.

## 5. Discussion

We have characterized the 50 mm gage two ways: i) by using direct dimensions of piston and cylinder and ii) by using dimensions of the cylinder and the Heydemann-Welch method to determine the clearance. Although the disagreement (7 × 10^−6^ at 170 kPa) between the effective areas for the two methods was within twice their combined standard uncertainties this was larger than we had hoped it would be [2 × 10^−6^ (*k* = 1)].

We believe that a possible explanation for the disagreement is that although the piston is very round, the cylinder is not as round as it could be. Because of this asymmetry the clearance between the piston and cylinder is not uniform and any interpretation of results based on flow through the crevice, which is the case with the H-W method, might be affected. We have modeled the flow of gas through a distorted cylinder and have found that the H-W method tends to substantially overestimate the clearance in cases in which the crevice does not have azimuthal symmetry.

It appears likely that the initial fabrication of the bare tungsten-carbide cylinder produced a round entity. However, the subsequent attachment of a separate cap to the tungsten carbide cylinder, which is necessary to allow it to function as a dead-weight tester, may have caused the distortion. The cylinder, comprised of two pieces (a titanium cap and a tungsten-carbide cylinder), appears to have roundness deviations of ± 300 nm as indicated by dimensional measurements performed at NIST. Additional roundness measurements were performed by NPL-India. Those measurements indicated that both the piston and cylinder were round ± 100 nm. We do not know the source of this disagreement.

A list of uncertainties for the effective areas for both characterizations is given in Table 3. Four of these contributions are common to both characterizations. These are the uncertainty of the area of the cylinder, *u*(*A*_0c_), the uncertainty of the thermal expansion coefficient, *u*(*α*), the uncertainty of the temperature, *u*(*T*) and the uncertainty of the cylinder’s pressure coefficient, *u*(*b*_c_). These are shown only once in Table 3 in order to avoid double counting of correlated uncertainties in the combined result. The thermal expansion coefficient was measured in our laboratory with a controlled environmental chamber and was found to be *α* = (9.06 ± 0.04) × 10^−6^/K. The uncertainty analysis of the dimensional results for the characterization yields a relative uncertainty of about 2.3 × 10^−6^ at 23 °C (*k* = 1). This seems slightly optimistic in view of results of the H-W analysis, which yields a result that differs by more than the combined standard uncertainty of the two methods. The uncertainty analysis of the H-W characterization of the clearance yields a relative uncertainty of about 3.8 × 10^−6^, (*k* = 1). In order to facilitate comparisons with other gages we will use the dimensional measurement results for the effective area but increase the uncertainty to include the discrepancy between the two measurements of the clearance. The overall uncertainty is obtained by the following formula:

$$\begin{array}{l} u(\bar{A})/\bar{A}=\sqrt{{\left(\frac{u(A_{\text{Dim}})}{A}\right)}^{2}+{\left(\frac{h_{\text{HW}}-h_{\text{Dim}})}{R\sqrt{3}}\right)}^{2}}\\ \;=\sqrt{2.3^{2}{\left(\frac{4.1+17P/\text{MPa}}{\sqrt{3}}\right)}^{2}}\times 10^{-6},\\ \;=(3.3+7.4P/\text{MPa}\times 10^{-6});(k=1). \end{array}$$ (22)

In Eq. (22) the uncertainty from the dimensional measurement is root sum squared with the difference between the clearance determined from the Heydemann-Welch method and the clearance determined from dimensional measurements, (*h*_HW_ − *h*_Dim_)/*R*, to allow for the fact that the two results did not overlap at the *k* = 1 level.

Lastly, some researchers have suggested the presence of a “gas species” or “crevice” effect that may be present in piston gauges because of viscosity differences as the gas flows through the annular region between the piston and cylinder [16]. We have attempted estimates of the magnitude of this effect, which may or may not have an effect on the area [17]. If present in this gauge the relative effect could be as high as 2.5 × 10^−6^ in gauge mode. Rather than systematically shift the area to account for this effect, we have chosen to increase the uncertainty by a small amount using a square distribution to cover it. The total uncertainty becomes:

$$u_{\text{tot}}(A)/A=4.1\times 10^{-6}+6.8\times 10^{-12}P/\text{Pa},\;(k=1).$$ (23)

## 6. Summary

We have investigated the performance of a controlled-clearance primary dead-weight tester designed for operation over a pressure range 35 kPa to 175 kPa. The gauge has a nominal diameter of 50 mm, which is large enough to facilitate good dimensional measurements not only of the piston but also the cylinder, which would normally go unmeasured. From the dimensional measurements and estimates of the pressure coefficients, a value is found for the effective area at 23 °C:

$$A_{\text{eff}}(P,P_{J}=1961.1192{\text{mm}}^{2}\times (1+3.75\times 10^{-12}P/\text{Pa}+3.05\times 10^{-12}P_{J}/\text{Pa}),$$ (24)

with an uncertainty *u*(*A*_eff_)/*A*_eff_ ≈ 2.3 ×10 ^−6^(1*σ*).

The piston/cylinder clearance was also characterized using the Heydemann-Welch method, which has traditionally been used at NIST and at other metrological institutions. This method implies a value that differs from the dimensional value by about 4 × 10^−6^ to 7 × 10^−6^ in the effective area. Taking this into account yields a total relative uncertainty in the effective area of (3.3+7.4 × 10^−6^*P*/Pa) × 10^−6^, (1*σ*). Allowing for the possibility of a 2.5 × 10^−6^ “gas species” or “crevice” effect [17] increases the total relative uncertainty to 4.1 × 10^−6^ + 6.8 × 10^−12^ *P*/Pa.

Auxiliary estimates of the clearance between the piston and cylinder were made using capacitance measurements. These were compared with estimates based on fall-rates and other estimates based on direct dimensional measurement. There is a variation δ*h/R* between these estimates of about 10 × 10^−6^. We believe that a significant contribution toward the variation of estimates for the clearance, and also of the estimates for the effective area is a result of the non-circularity of the cylinder. As of this writing the cap associated with the cylinder has been refitted. Preliminary analyses of new dimensional measurements are being performed at this time.

## Figures and Tables

**Table 1:** Fall-rate measurements with N_2_

*P*_1_-*P*_0_ (kPa)	*P*_J_ (kPa)	F.R.^1/3^ (m/s)^1/3^
40.0	103.4	0.004233
40.0	103.4	0.004239
40.0	310.3	0.004066
40.0	293.0	0.004101
40.0	420.6	0.004000
40.0	568.8	0.003825
40.0	558.5	0.003861
40.0	620.5	0.003803
40.0	689.5	0.003756
40.0	723.9	0.003723
40.0	758.4	0.003692
70.0	189.6	0.004868
70.0	241.3	0.004807
70.0	275.8	0.004778
70.0	344.7	0.004708
70.0	448.2	0.004629
70.0	551.6	0.004525
70.0	655.0	0.004430
70.0	723.9	0.004373
70.0	792.9	0.004309
105.0	137.9	0.005714
105.0	241.3	0.005582
105.0	448.2	0.005393
140.0	310.3	0.006214
140.0	448.2	0.006057
140.0	586.1	0.005949
140.0	723.9	0.005784
140.0	827.4	0.005686
140.0	958.4	0.005582
140.0	999.7	0.005579
140.0	1103.0	0.005479
140.0	1241.0	0.005291
140.0	1379.0	0.005167
140.0	1517.0	0.005035
140.0	1586.0	0.004984
140.0	1655.0	0.004888
140.0	1793.0	0.004721
177.4	206.8	0.006751
177.4	344.7	0.006586
177.4	551.6	0.006390
177.4	689.5	0.006278
177.4	896.3	0.006020
177.4	1069.0	0.005864
177.4	1241.0	0.005739
177.4	1344.0	0.005637
177.4	1465.0	0.005479
177.4	1586.0	0.005364
177.4	1655.0	0.005296
177.4	1913.0	0.005026

**Table 2:** “d” measurements—(−1/*A*)d*A*/d*P*_J_

Load, (N)	Pressure, (kPa)	“*d*”×10^12^, (Pa^−1^)
139	70.88	−3.75
139	70.88	−3.80
139	70.88	−3.85
139	70.88	−3.90
139	70.88	−3.94
206	105.05	−3.82
206	105.05	−3.84
206	105.05	−3.86
206	105.05	−3.88
206	105.05	−3.89
206	105.05	−3.91
274	139.72	−3.81
274	139.72	−3.81
274	139.72	−3.83
274	139.72	−3.87
274	139.72	−3.90
274	139.72	−3.91
348	177.46	−3.77
348	177.46	−3.8
348	177.46	−3.81
348	177.46	−3.82
348	177.46	−3.82
348	177.46	−3.83
348	177.46	−3.86
348	177.46	−3.86

**Table 3:** Comparison of fractional uncertainties for two characterizations

Component	Value	Contribution to *u*(*A*_eff_)/*A*_eff_×10^6^	
		Method 1 Dim. unc.	Method 2 H-W unc.
*u*(*A*_0p_)/*A*_0p_	2.1 × 10^−6^	2.1/2	
*u*(*A*_0c_)/(*A*_0c_	2.5 × 10^−6^	2.5/2	
Δ*T* × *u*(*α*)	3 K × 0.04 × 10^−6^/K	0.12	
*α* × *u*(*T*)	9.06 × 10^−6^ K^−1^ × 0.05 K	0.45	
*P* × *u*(*b*_p_)	*P* × 0.17 × 10^−12^Pa^−1^	0.17 × 10^−6^ *P* / Pa	
*P* × *u*(*b*_c_)	*P* × 0.55 × 10^−12^Pa^−1^	0.55 × 10^−6^ *P* / Pa	
*P*_J_ × *u*(*b*_J_)	*P*_J_^−1^ × 0.3 × 10^−12^Pa	0.3 × 10^−6^ *P*_J_ / Pa	
*d* × *u*(*P*z)	3.98 × 10^−12^Pa^−1^ × 0.82 × 10^6^Pa		3.3
(*P*_Z_ – *P*_J_ × *u*(*d*)	(6.0 × 10^6^ Pa – *P*_J_) 0.3× 10^−12^Pa^−1^		1.8 – 0.3 × 10^−6^ *P*_J_ / Pa

Each method	*u*(*A*)/*A*) × 10^6^	~2.3		~3.8
Combined	*u*_1,2_(*A*)/*A*) × 10^6^		~4.2	
Possible crevice effect (Type B)	*u*_CE_(*A*)/*A*) × 10^6^		~1.44	

Total	*u*_Tot_(*A*)/*A*) × 10^6^		~4.4	(*k* = 1)

## Footnotes

1 Certain commercial equipment, instruments, or materials are identified in this paper to foster understanding. Such identification does not imply recommendation or endorsement by the National Institute of Standards and Technology, nor does it imply that the materials or equipment identified are necessarily the best available for the purpose.

## References

[1] Guildner LA, Stimson HF, Edsinger RE, Anderson RL (1970). Metrologia.

[2] Tilford CR, Hyland RW, CR Tilford (1988). Proc XI IMEKO World Congress, Houston, Texas, 1988.

[3] Welch BE, Edsinger RE, Bean VE, Ehrlich CD, Molinar GF (1989). High Pressure Metrology. Bureau International des Poids et Mesures Monographie.

[4] Heydemann PLM, Tilford CR, Hyland RW (1977). J Vac Sci Technol.

[5] Johnson DP, Newhall DH (1953). The Piston Gage as a Precise Pressure-Measuring Instrument. Transactions of the ASME.

[6] Heydemann PLM, Welch BE, LeNeindre B, Vodar B (1975). Experimental Thermodynamics.

[7] Westergaard HM (1952). Chap. V in Theory of Elasticity and Plasticity Cambridge.

[b8-j80kam] 8R. P. Singhal of the National Physical Laboratory-India (private communication).

[b9-j80kam] 9J. Stoup, NIST Precision Engineering Divison calibration reports a) M5238-1, 15 November 1996 and b) M5901, 26 August 1998.

[b10-j80kam] 10J. L. M. Poiseuille (1840).

[11] Landau LP, Lifshitz EM (1959). Fluid Mechanics.

[12] Molinar GF, Vitasso M (1976). High Temp. High Press.

[13] Dolinskii EF (1972). Loskutov, Polukhin, Measurement Techniques.

[14] Meyers CH, Jessup RS (1931). J Res Natl Bur Stand (US).

[15] Reitz JR, Milford FJ (1967). Foundations of Electromagnetic Theory.

[16] Tilford CR, Hyland RW, Yi-Tang S (1989). High Pressure Metrology. BIPM Monographie.

[17] Schmidt JW, Cen Y, Driver RG, Bowers WJ, Houck JC, Tison SA, Ehrlich CD (1999). Metrologia.

